# Using Platelet Parameters to Anticipate Morbidity and Mortality Among Preterm Neonates: A Retrospective Study

**DOI:** 10.3389/fped.2020.00090

**Published:** 2020-03-13

**Authors:** Hayato Go, Hitoshi Ohto, Kenneth E. Nollet, Shunya Takano, Nozomi Kashiwabara, Mina Chishiki, Hajime Maeda, Takashi Imamura, Yukihiko Kawasaki, Nobuo Momoi, Mitsuaki Hosoya

**Affiliations:** ^1^Department of Pediatrics, Fukushima Medical University School of Medicine, Fukushima, Japan; ^2^Department of Advanced Cancer Immunotherapy, Fukushima Medical University School of Medicine, Fukushima, Japan; ^3^Department of Blood Transfusion and Transplantation Immunology, Fukushima Medical University School of Medicine, Fukushima, Japan; ^4^Department of Pediatrics, Sapporo Medical University School of Medicine, Sapporo, Japan

**Keywords:** platelet parameters, mean platelet volume, premature neonates, plateletcrit, mortality

## Abstract

**Background:** Platelets participate in many physiological and pathological functions and some platelet parameters predict adult diseases. However, few studies report whether platelet parameters may reflect neonatal disease and mortality in a large cohort.

**Objective:** We aimed to investigate whether platelet parameters could predict bronchopulmonary dysplasia (BPD), necrotizing enterocolitis (NEC), intraventricular hemorrhage (IVH), and NICU mortality.

**Study Design and Methods:** This retrospective cohort study examined records from 2006 to 2017 at the neonatal intensive care unit (NICU) of Fukushima Medical University Hospital. We retrospectively investigated platelet count, plateletcrit (PCT), mean platelet volume (MPV), and platelet distribution width (PDW) on the first day of life in preterm newborns born <32 weeks' gestation admitted to our NICU from 2006 to 2017. Receiver operating characteristic (ROC) and multiple regression analyses, along with Cox proportional hazard modeling, identified independent predictors of morbidities and mortality in preterm newborns.

**Results:** Of 1,501 neonates admitted to our NICU, a total of 305 preterm newborns were included in this study. Gestational age, birth weight, and Apgar score were significantly lower in non-survivors than in survivors. Platelet count, PCT, PDW and PMI did not differ significantly between the two groups, whereas mean MPV in non-survivors was significantly higher than in survivors (10.5 fl vs. 10.0 fl, *p* = 0.001). Multivariate Cox hazard modeling showed that shorter GA [HR: 0.628, 95% CI: 0.464–0.840, *p* = 0.003], male sex [HR: 0.269, 95% CI: 0.113–0.640, *p* = 0.001], and MPV [HR: 1.469, 95% CI: 1.046–2.063, *p* = 0.026] independently predicted overall survival. Per receiver operating curve, an MPV threshold of 10.2 fl. MPV predicts prognosis in neonates with a sensitivity of 72.4% and a specificity of 58.6% (AUC = 0.685, 95% CI: 0.600–0.789, *p* = 0.001). Furthermore, multivariate analysis revealed that platelet parameters were not associated with BPD and NEC, whereas small for gestational age (SGA), Apgar score at 5 min, and low PCT were associated with intraventricular hemorrhage (IVH).

**Conclusion:** This study demonstrates that low PCT predicts IVH, and MPV ≥ 10.2 fL correlates with mortality among infants born after <32 weeks' gestation.

## Introduction

Platelets participate in many physiological and pathological functions and some platelet parameters predict adult diseases (1–3). High mean platelet volume (MPV) values are associated with coronary flow and high risks of recurrent stroke in adults (4, 5). Elevated MPV, platelet distribution width (PDW), and increased plateletcrit (PCT) are associated with mortality in diseases such as ischemic coronary artery disease, pneumonia, kidney disease, and cancer (6–9). Platelet activation and consumption are seen commonly in critical ill patients and indicate a poorer prognosis (10). MPV and PDW are easy-to-measure surrogates for platelet activation that is provoked inflammation. In particular, MPV can reflect platelet function better than a platelet count by itself. Likewise, previous studies have implicated high MPV in the first postpartum hours with increased risk of necrotizing enterocolitis (NEC), bronchopulmonary dysplasia (BPD), and intravascular hemorrhage (IVH) in neonates (11, 12). On the other hand, platelet mass index (PMI), calculated by multiplying platelet count by MPV, is associated with retinopathy of prematurity, NEC, and IVH (13). However, few studies report whether platelet parameters (MPV, PDW, and PMI) may reflect neonatal disease such as BPD, NEC, and IVH in a large cohort. Furthermore, it has not been established whether MPV, PDW, or PMI correlate with mortality in preterm infants. The primary objective of this study was to evaluate the potential of platelet parameters to predict BPD, IVH, and NEC in preterm infants. The secondary objective was to evaluate the associations between platelet parameters and mortality.

## Methods

### Study Design and Population

This retrospective cohort study examined records from 2006 to 2017 at the neonatal intensive care unit (NICU) of Fukushima Medical University Hospital (FMU). The Ethics Committee of FMU, guided by local policy, national law, and the World Medical Association Declaration of Helsinki, approved this study without requiring informed consent from guardians. Neonates born <32 weeks' gestation were included in this study. Exclusion criteria were congenital anomalies and neonates not tested for platelet parameters within 12 h of birth.

### Definition of BPD, IVH, and NEC

BPD was defined as the need for supplemental oxygen or supplemental ventilation at 36 weeks' postmenstrual age (14). IVH was defined on the basis of ultrasound scans on postnatal days 1, 4, 7, 14, 21, and 28, using Papille's classification (15). NEC was staged according to Bell's criteria, with pneumatosis intestinalis, hepatobiliary gas, and free intraperitoneal air on radiography as major factors (16).

### Platelet Parameter Measurements

Blood samples were collected through umbilical cord or peripheral venipuncture to measure the PCT, MPV, PDW, and PMI of each newborn. Complete blood counts were measured using a Sysmex CS-5100 coagulation analyzer (Sysmex, Kobe, Japan) on admission. PMI was calculated as the product of platelet count and MPV.

### Prenatal and Postnatal Risk Factors

Platelet parameters were compared with demographic variables including gender, birth weight, and gestational age. Furthermore, we considered pregnancy-induced hypertension (PIH); chorioamnionitis (CAM); preterm rupture of membranes (PROM); hemolysis, elevated liver enzymes, and low platelet count (HELLP syndrome); and placental abruption (PA) as possible prenatal risk factors, and small for gestational age (SGA, birth weight <10th percentile), respiratory distress syndrome (RDS), and Apgar score as factors affecting platelet parameters.

### Statistical Analysis

Platelet parameters from medical records were rendered as median interquartile range (IQR) percentiles for continuous variables of non-normal distribution. Differences in categorical variables and continuous variables were assessed for significance using Chi-square and Mann-Whitney U-tests, respectively. The 1-year overall survivals were plotted on Kaplan-Meier curves, with differences assessed for significance by log-rank test. Correlations with *p* < 0.05 by univariable analysis were entered into a multivariable Cox proportional hazard regression model or multiple regression analysis to identify independent prognostic factors. A receiver operating characteristic (ROC) curve identified predictive MPV levels, along with their sensitivity and specificity.

SPSS for Mac, release 25.0 (SPSS, Chicago, IL) was used to perform the statistical analyses with *P* < 0.05 considered to be statistically significant.

## Results

**Figure 1:**
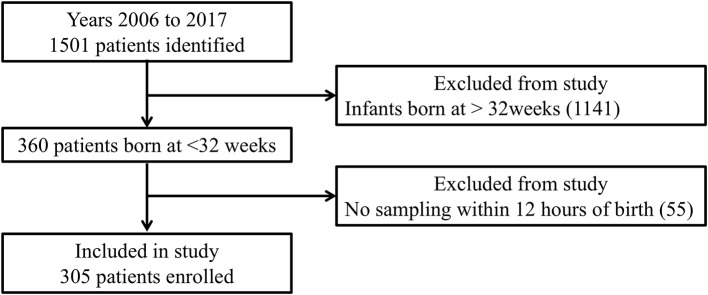
Flow chart of this study.

Of 1,501 neonates admitted to our NICU between January 2006 and December 2017, there were 305 born at <32 weeks' gestation who were assessed within 12 h postpartum (Figure 1). BPD, IVH, and NEC were diagnosed in 76, 54, and 10 of them, respectively. As shown in Table 1A, multivariate analysis showed that shorter GA was significantly correlated with BPD. Furthermore, as shown in Table 1B, SGA, lower PCT, and lower Apgar scores at 5 min were significantly correlated with IVH. In contrast, Table 1C shows no significant factors affecting NEC.

**Table 1:** The relationships between morbidities and clinical characteristics and platelet parameters in preterm newborns.

	**BPD (*n* = 76)**	**non BPD (*n* = 229)**	**Univariable analysis**	**Multivariable analysis**
**(A) Preterm newborns born** ** <32 weeks with BPD vs. without BPD**				
GA (weeks)	24.9	28.5	0.000	0.000
BW (g)	632	1012	0.000	0.294
CAM *n* (%)	41 (51)	66 (24)	0.000	0.929
PROM *n* (%)	29 (35)	46 (17)	0.000	0.374
RDS *n* (%)	88 (69)	91 (51)	0.001	0.919
Apgar Score 1 min	4.5	5	0.000	0.786
Apgar Score 5 min	6.5	8	0.000	0.634
MPV (fl)	10.4	9.9	0.000	0.063
	**IVH (*****n=*** **54)**	**non IVH (*****n=*** **251)**	**Univariable analysis**	**Multivariable analysis**
**(B) Preterm newborns born** ** <32 weeks with IVH vs. without IVH**				
SGA *n* (%)	20 (37)	37 (15)	0.000	0.002
Apgar Score 1 min	4	5	0.021	0.318
Apgar Score 5 min	7	8	0.001	0.002
Platelet counts (1,000/mm^3^)	16.7	21.7	0.005	0.833
PCT (%)	0.16	0.21	0.002	0.021
PMI (fl nl^−1^)	1654	2044	0.012	0.105
	**NEC (*****n=*** **10)**	**non NEC (*****n=*** **295)**	**Univariable analysis**	**Multivariable analysis**
**(C) Preterm newborns born** ** <32 weeks with NEC vs. without NEC**				
GA (weeks)	25.3	27.4	0.008	0.337
Apgar Score 1 min	3	5	0.028	0.286

*BPD, bronchopulmonary dysplasia; GA, gestational age; BW, birth weight; RDS, respiratory distress syndrome; CAM, chorioamnionitis; ROM, preterm rupture of membranes; RDS, respiratory distress syndrome; MPV, mean platelet volume; IVH, intraventricular hemorrhage; PCT, plateletcrit; PMI, Platelet mass index; NEC, necrotizing enterocolitis*.

Table 2 describes non-survivors and survivors. Regrettably, 31 were non-survivors. Among non-survivors, gestational age, birth weight, and Apgar score were significantly lower than in survivors. Platelet counts, PCT, PDW and PMI were not significantly different between the two groups, whereas MPV in non-survivors was significantly higher than in survivors (10.5 vs. 10.0, *p* = 0.001) (Table 2).

**Table 2:** Characteristics of survivors and non-survivors enrolled in the study.

		**Non-Survivors (*n =* 31)**	**Survivors (*n =* 274)**	***P*-value**
Neonatal factor	GA (w) (median) [IQR]	24.9 [23.5–26.4]	27.7 [25.5–30.0]	<0.001
	BW (g) (median) [IQR]	598 [534–830]	937 [693–1,220]	<0.001
	Male *n* (%)	24 (77)	130 (47)	0.002
	RDS *n* (%)	17 (55)	163 (59)	0.618
	SGA *n* (%)	9 (29)	48 (18)	0.119
	IVH *n* (%)	7 (23)	47 (17)	0.453
	BPD *n* (%)	9 (30)	61 (22)	<0.001
	NEC *n* (%)	4 (13)	6 (2)	0.001
	Apgar Score (1 min) (median) [IQR]	3 [2–5]	5 [3–7]	<0.001
	Apgar Score (5 min) (median) [IQR]	6 [4–7]	8 [6–8]	0.001
	Platelet counts (×10^4^/μl) (median) [IQR]	19.8 [13.3–26.9]	20.9 [15.5–25.9]	0.555
	PCT (%) (median) [IQR]	0.20 [0.14–0.26]	0.20 [0.15–0.24]	0.991
	MPV (fl) (median) [IQR]	10.5 [10.0–11.2]	10.0 [9.2–10.5]	0.001
	PDW (%) (median) [IQR]	12.2 [10.7–13.7]	11.5 [10.4–13.9]	0.275
	PMI (fl nl^−1^) (median) [IQR]	2,149 [1,438–2,632]	2,028 [1,512–2,429]	0.876
Maternal factor	PROM *n* (%)	11 (35)	64 (23)	0.137
	CAM *n* (%)	16 (52)	91 (33)	0.056
	PA *n* (%)	7 (23)	28 (10)	0.042
	PIH *n* (%)	6 (19)	29 (11)	0.146
	HELLP *n* (%)	2 (6)	9 (3)	0.370
	GDM n (%)	0 (0)	7 (3)	0.327

*GA, gestational age; BW, birth weight; RDS, respiratory distress syndrome; SGA, small for gestational age; IVH, intraventricular hemorrhage; BPD, bronchopulmonary dysplasia; NEC, necrotizing enterocolitis; PCT, plateletcrit; MPV, mean platelet volume; PDW, platelet distribution width; PMI, Platelet mass index; PROM, preterm rupture of membranes; CAM, chorioamnionitis; PA, placental abruption; PIH, pregnancy-induced hypertension; HELLP, hemolysis, elevated liver enzymes, low platelets; GDM, gestational diabetes mellitus; IQR, interquartile range. Continuous variables are presented as medians*.

**Figure 2:**
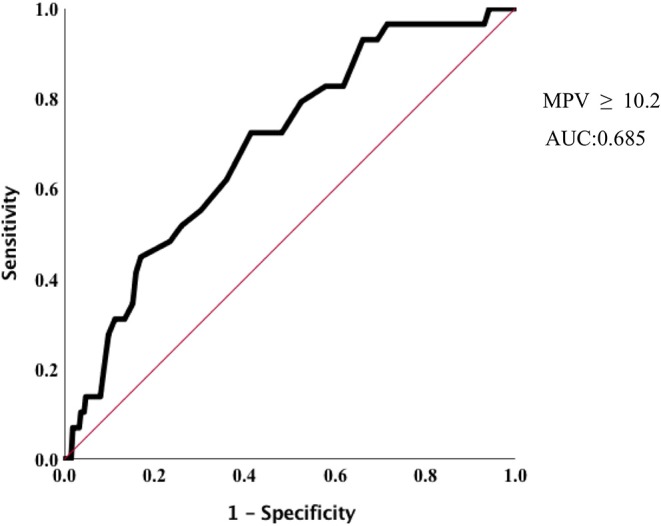
Optimized cut-off value was determined for MPV using standard ROC curve.

**Figure 3:**
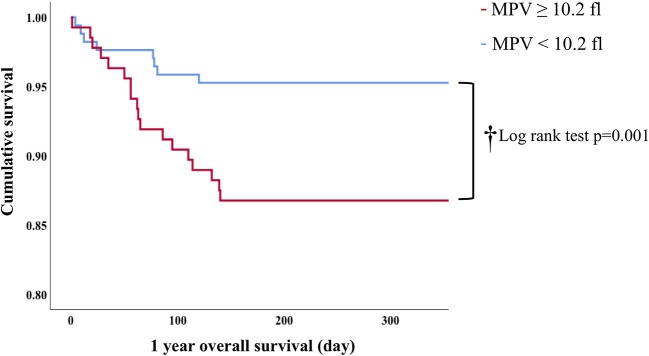
Kaplan-Meier analysis of 1-year overall survival in preterm infants.

In Cox proportion univariable analysis, shorter GA, smaller BW, male sex, presence of PA and/or BPD, lower Apgar score, and MPV were significant predictors of overall survival (Table 3). Multivariable Cox hazard modeling showed that shorter GA [HR: 0.628, 95% CI: 0.464–0.840, *p* = 0.003], male sex [HR: 0.269, 95% CI: 0.113–0.640, *p* = 0.001], and MPV [HR: 1.469, 95% CI: 1.046–2.063, *p* = 0.026], independently predicted overall survival (Table 3). The ROC curve comprising Figure 2 shows than an MPV ≥ 10.2 fl is prognostic with a sensitivity of 72.4% and a specificity of 58.6% (AUC = 0.685, 95% CI: 0.600–0.789, *p* = 0.001). Thus, newborns were divided into two groups: MPV ≥ 10.2 fl (*n* = 136) and MPV < 10.2 fl (*n* = 169). The Kaplan-Meier curves of preterm neonates with MPV ≥ 10.2 and MPV < 10.2 were significantly different (Figure 3). As shown in Table 4, those with MPV ≥ 10.2 fl were lower in GA, BW, Apgar score and platelet counts; were higher in PDW; and had higher incidence of CAM and BPD. To further advance the prognostic value of MPV, we performed univariate analysis and multivariate Cox proportional hazard modeling to establish hazard ratios (HR).

**Table 3:** The relationship between clinical parameters and MPV levels in preterm newborns.

		**MPV ≥ 10.2 (*n =* 136)**	**MPV < 10.2 (*n =* 169)**	***P*-value**
Neonatal factor	GA (w) (median) [IQR]	26.5 [24.3–29.1]	28.0 [25.9–30.2]	0.002
	BW (g) (median) [IQR]	739 [596–1,071]	1,010 [771–1,279]	0.000
	Male *n* (%)	69 (50)	83 (49)	0.386
	RDS *n* (%)	81 (60)	99 (59)	0.430
	SGA *n* (%)	29 (21)	28 (17)	0.253
	BPD *n* (%)	48 (35)	28 (17)	0.000
	IVH *n* (%)	25 (18)	29 (17)	0.429
	NEC *n* (%)	5 (3)	5 (3)	0.477
	Death *n* (%)	22 (16)	9 (5)	0.002
	Apgar Score (1 min) (median) [IQR]	4 [3–6]	5 [3.5–7]	0.026
	Apgar Score (5 min) (median) [IQR]	7 [6–8]	8 [6–8]	0.048
	Platelet counts (×10^4^/μl) (median) [IQR]	18.6 [13.8–24.1]	22.4 [16.8–26.3]	0.001
	PCT (%) (median) [IQR]	0.20 [0.15–0.26]	0.19 [0.15–0.24]	0.246
	PDW (%) (median) [IQR]	11.9 [11.2-13.2]	10.8 [10.1–15.9]	0.000
	PMI (fl nl^−1^) (median) [IQR]	2,055 [1,516–2,555]	1,953 [1,489–2,407]	0.313
Maternal factor	PROM *n* (%)	35 (26)	69 (40)	0.366
	CAM *n* (%)	61(46)	47 (28)	0.001
	PA *n* (%)	13 (10)	13 (8)	0.230
	PIH *n* (%)	14 (10)	25 (15)	0.111
	HELLP *n* (%)	8 (2)	9 (5)	0.570
	GDM *n* (%)	3 (2)	4 (2)	0.233

*GA, gestational age; BW, birth weight; RDS, respiratory distress syndrome; SGA, small for gestational age; IVH, intraventricular hemorrhage; BPD, bronchopulmonary dysplasia; NEC, necrotizing enterocolitis; PCT, plateletcrit; MPV, mean platelet volume; PDW, platelet distribution width; PMI, Platelet mass index; PROM, preterm rupture of membranes; CAM, chorioamnionitis; PA, placental abruption; PIH, pregnancy-induced hypertension; HELLP, hemolysis, elevated liver enzymes, low platelets; GDM, gestational diabetes mellitus; IQR, interquartile range. Continuous variables are presented as medians*.

**Table 4:** Univariable and multivariable analysis overall survival in newborns <32 weeks.

	**Univariable analysis**			**Multivariable analysis**		
	**Hazard ratio**	**95% CI**	***P*-value**	**Hazard ratio**	**95% CI**	***P*-value**
GA	0.689	0.581–0.818	0.000	0.628	0.464–0.840	0.003
BW	0.998	0.996–0.999	0.001	1.001	0.999–1.003	0.407
Male	0.282	0.121–0.654	0.003	0.269	0.113–0.640	0.003
RDS	1.184	0.584–2.401	0.640	–	–	–
SGA	0.548	0.252–1.190	0.129	–	–	–
IVH	0.709	0.306–1.646	0.424	–	–	–
BPD	0.196	0.094–0.409	0.010	1.546	0.659–3.627	0.099
NEC	0.177	0.062–0.506	0.001	0.509	0.151–1.710	0.275
Apgar Score (1 min)	0.721	0.607–0.856	0.000	0.880	0.678–1.143	0.129
Apgar Score (5 min)	0.756	0.653–0.875	0.000	0.971	0.773–1.221	0.675
Platelet counts	0.992	0.945–1.041	0.750	–	–	–
PCT	5.077	0.049–607.8	0.506	–	–	–
MPV	0.315	0.145–0.684	0.003	0.441	0.136–2.063	0.026
PDW (%)	1.030	0.925–1.167	0.520	–	–	–
PMI (fl nl^−1^)	1.000	1.000–1.001	0.412	–	–	–
PROM	0.579	0.278–1.209	0.146	–	–	–
CAM	0.508	0.251–1.027	0.059	–	–	–
PA	0.420	0.181–0.976	0.044	0.438	0.163–1.177	0.102
PIH	0.532	0.218–1.296	0.165	–	–	–
HELLP	0.516	0.123–2.165	0.366	–	–	–
GDM	0.047	0.465–2.104	0.547	–	–	–

*GA, gestational age; BW, birth weight; RDS, respiratory distress syndrome; SGA, small for gestational age; IVH, intraventricular hemorrhage; BPD, bronchopulmonary dysplasia; NEC, necrotizing enterocolitis; PCT, plateletcrit; MPV, mean platelet volume; PDW, platelet distribution width; PMI, Platelet mass index; PROM, preterm rupture of membranes; CAM, chorioamnionitis; PA, placental abruption; PIH, pregnancy-induced hypertension; HELLP, hemolysis, elevated liver enzymes, low platelets; GDM, gestational diabetes mellitus. Continuous variables are presented as medians*.

## Discussion

The present study shows that elevated MPV at birth correlates with unfavorable outcomes for NICU patients born after <32 weeks' gestation. While precise pathophysiologic mechanisms remain elusive, platelet numbers and average size may increase in conjunction with inflammatory and thrombotic conditions (17). Young platelets are generally larger than old ones, and the presence of more young platelets indicates increased production in response to consumption, which in turn may be provoked by inflammation. Thus, an elevated MPV may be indicative of oxidative stress in newborns (18). In short, MPV in preterm newborns can inform clinicians of possible hypercoagulative states, increased inflammatory response, and oxidative stress. Among these, a possible explanation for the relationship between MPV and mortality is inflammatory response. It is known that inflammatory response is significantly associated with adverse clinical outcomes in ICU patients. Platelet parameters such as MPV are immediate indicators of platelet activation that is driven by inflammatory processes.

In adults, some studies showed that the initial MPV, as a predictor of mortality in critically ill patients, was not significantly associated with in-hospital mortality (19), although other studies showed that elevated MPV during hospitalization was a predictor of mortality in critically ill adults (9, 20). High MPV was suggested to be associated with overall survival in various diseases such as cancer, cardiac disease, septic shock, kidney injury, and patients hospitalized in a respiratory intensive care unit (21–25). On the other hand, subsequent MPV and PDW are suggested to be associated with mortality in critically ill children receiving mechanical ventilation, however initial admission MPV was not associated with mortality in those children (17).

There are few reports about the relationship between MPV and morbidities among large cohorts of premature newborns. Cekmez et al. (11) found that elevated MPV measured within 2 h of birth was associated with BPD, IVH, and NEC using univariate analysis in 44 BPD, 42 IVH, and 21 NEC preterm newborns, but they did not perform multivariate analysis. In our study, univariate analysis also suggested that MPV at birth was higher in BPD vs. non-BPD, but multivariate analysis showed no relationship between BPD and MPV. On the other hand, Dani et al., also suggested that elevated MPV at 24 to 48 h of life was higher in BPD than in non-BPD groups, however, there was no significant difference of MPV between IVH and non-IVH groups (12). Korkmaz et al. also suggested that platelet counts, MPV, and PMI at 12 h postpartum were not associated with IVH (26). In this study, PCT is an independent risk factor for IVH. To date, previous research suggested that PCT had a predictive value for RDS in preterm newborns (27). However, few studies investigated the relationship between PCT and IVH. Further studies are needed to investigate the mechanism(s) relating PCT with IVH.

Our study has several limitations. First, we could not assess the changes of MPV values during early postnatal life. Previous studies reported that BPD correlated with MPV changes at 24 to 48 h of life. However, to avoid phlebotomy, we did not perform complete blood counts at 48 h. Second, we could not investigate the relationship between platelet indices and sepsis because few of our preterm infants had sepsis at birth. Other studies associated sepsis with elevated MPV in neonates (28–30). Third, an interesting finding in our study is that there is no association between MPV and preterm morbidities (BPD/NEC) that might be related to inflammation or oxidative stress, only with mortality. We could not elucidate any pathologic mechanism of this phenomenon. Some studies also suggest that among critically ill patients, including children and adults, higher MPV is strong predictor of mortality. However, mechanisms have not been elucidated. Our study is limited by its retrospective nature and the lack of clear causality between platelet parameters and mortality. Future prospective studies and multicenter collaboration may be needed to address this relationship.

Finally, we could not assess the relationship between serum lactate at birth and subsequent mortality. While there are missing data in this study, lactate and pH values in cord or venous blood at birth were not associated with mortality (not shown). Some studies reported that high lactate levels and low pH in cord blood within the first day of life predict mortality in premature infants (31, 32). Conversely, other studies found no association between umbilical cord acidemia and adverse outcomes in those born at <32 weeks (33, 34). Gaudier et al., reported that 1 and 5 min Apgar scores were better predictors of survival than umbilical artery blood gases in 1,073 infants weighing 500–1,000 g at birth (35). However, in this study, no correlation emerged between mortality and Apgar scores. Wedzicha et al. reported that there was a negative correlation between MPV and PaO_2_ (*r* = −0.70) in patients with chronic air-flow obstruction (36). Thus, perinatal hypoxia may have a direct effect on platelet production and elevated MPV.

In conclusion, the present study demonstrates that MPV ≥ 10.2 fl at birth is an independent risk associated with neonatal mortality, and lower PCT was a predictor of IVH. The mechanisms behind the association between high MPV and mortality in preterm infants warrant further investigation.

## Data Availability Statement

The datasets analyzed in this article are not publicly available. Requests to access the datasets should be directed to gohayato2525@gmail.com.

## Ethics Statement

The studies involving human participants were reviewed and approved by Institution of the ethics committee of Fukushima Medical University. Written informed consent to participate in this study was provided by the participants' legal guardian/next of kin.

## Author Contributions

HG and HO conceived and coordinated the study and wrote the paper. HG designed the research. HG, MC, NK, ST, TI, HM, NM, YK, and MH collected and analyzed the data. KN and HG edited the manuscript and provided general guidance. All authors reviewed the results and approved the final version of the manuscripts.

### Conflict of Interest

The authors declare that the research was conducted in the absence of any commercial or financial relationships that could be construed as a potential conflict of interest.
